# Increased GABA signaling in liver macrophage promotes HBV replication in HBV-carrier mice

**DOI:** 10.1016/j.virusres.2024.199366

**Published:** 2024-03-30

**Authors:** Yunling Chen, Zhaoqing Yin, Xiaonan Zhang, Yiwei Zhao, Tinghao Liu, Wei-Yang Lu, Shuanglian Wang

**Affiliations:** a Medical Science and Technology Innovation Center, Shandong First Medical University & Shandong Academy of Medical Sciences, Jinan, China; b Department of Physiology and Pathophysiology, School of Basic Medical Science, Shandong University, Jinan, China; c School of Clinical and Basic Medical Sciences, Shandong First Medical University& Shandong Academy of Medical Sciences, Jinan, China; d Department of Physiology and Pharmacology, Robarts Research Institute, University of Western Ontario, Canada

**Keywords:** GABA, macrophage, Macrophage polarization, HBV replication

## Abstract

Gamma-aminobutyric acid (GABA) signals in various non-neuronal cells including hepatocytes and some immune cells. Studies, including ours, show that type A GABA receptors (GABA_A_Rs)-mediated signaling occurs in macrophages regulating tissue-specific functions. Our recent study reveals that activation of GABA_A_Rs in liver macrophages promotes their M2-like polarization and increases HBV replication in mice. This short article briefly summarizes the GABA signaling system in macrophages and discusses potential mechanisms by which GABA signaling promotes HBV replication.

## Highlights

• A functional GABA signaling system exists in liver macrophages.
• Activation of GABA signaling leads to the M2-like anti-inflammatory phenotype of liver macrophages.
• GABA signaling activation promotes HBV replication in HBV-carrier mice.

## 1 Introduction

Gamma-aminobutyric acid (GABA) is a non-protein amino acid produced by various types of cells including vertebrate cells, as well as some gut bacteria and plant cells, where GABA is primarily produced by decarboxylation of glutamate via the catalytic activity of glutamic acid decarboxylase (GAD) (Fenalti et al., 2007). In mammalian cells, GABA generates biological signaling mainly through activation of its ionotropic type A or metabotropic type B receptors (GABA_A_Rs or GABA_B_Rs). GABA_A_Rs are heteropentameric Cl^−^ channels that are assembled diversely from 19 different subunits (α1-6, β1-3, γ1-3, δ, ε, π, θ, and ρ) (Zhu et al., 2018) . Activation of GABA_A_Rs in mature neurons induces Cl^−^ influx and membrane hyperpolarization and inhibition, thus GABA has long been known as the primary inhibitory neurotransmitters (Felmlee et al., 2021)..

As an effective immunomodulatory molecule (Bjurstöm et al., 2008; Jin et al., 2013), GABA induces signals in various non-neuronal cells including epithelial cells (Li et al., 2012; Xiang et al., 2007), macrophages (Bhandage and Barragan, 2021), lymphocytes (Dionisio et al., 2011; Mendu et al., 2012). The physiological and pathophysiological roles of GABA signaling in the regulation of cellular immunity have been widely investigated (Januzi et al., 2018; Kim et al., 2018; Xiang et al., 2007; Zhang et al., 2021). For example, the transition of monocytes to M1 macrophages involves changes both in GABA receptor subunits and the GABA synthesizing enzyme GAD, and this evidence suggests that GABA may play a critical role as modulators of the peripheral immune response (Ruiz-Rodriguez et al., 2023). However, the issue as to whether and how GABA signaling in macrophages influences antiviral host defenses remains poorly understood. This commentary briefly discusses how GABA signaling regulates the phenotypic development of macrophages, and discusses potential mechanisms by which GABA signaling promotes Hepatitis B virus (HBV) replication.

## 2 Increasing GABA promotes HBV replication in HBV-carrier mice

HBV can infect and replicate within hepatocytes and causes hepatitis, a very common chronic liver disease. Notably, HBV evade innate immunity of hepatocytes but activates macrophages during infection of the liver (Cheng et al., 2017). The adeno-associated virus transduction-mediated replicon delivery mouse model displays persistent HBV replication and induces specific immune response, and this mouse model is widely used for investigating host immune responses and for evaluating antiviral compounds (Du et al., 2021). This HBV-carrier mouse model is established by hydrodynamic injection of a total of 6 μg adeno-associated virus plasmids (pAAV/HBV1.2) diluted in saline with liquid equal to 8–10% mouse weight within 5–8 s. During this procedure, HBV DNA plasmids enter hepatocytes under the high pressure that permeabilizes the capillary endothelium and generates “pores” in the plasma membrane of surrounding hepatocytes, through which DNAs may reach the intracellular space. Then the retained HBV plasmid DNAs initiate HBV replication by transcription of pregenomic RNA and other HBV RNAs, followed by the formation of HBV replication intermediates and expression of HBV proteins (Du et al., 2021).

Given that high-concentration of GABA molecules in the intestine produced by bacteria (Otaru et al., 2021) and/or from foods (Briguglio et al., 2018), can be absorbed and then get into the liver through the portal vein, we recently studied how elevating GABA signaling affects HBV replication using an HBV-carrier mice model (Bao et al., 2023). The main findings from this study are summarized below.

### 2.1 Liver macrophages involve in the GABA_A_R-mediated increase of HBV replication

Using control and HBV-carrier mice, we demonstrated GABA_A_R-mediated signaling mechanism in liver macrophages including the residential Kupffer cells. Interestingly, the expression of GABA_A_R subunit in F4/80^+^ liver macrophages increased in HBV-carrier mice compared to control mice, suggesting that HBV replication in hepatocytes may alter GABA signaling in liver macrophages. To investigate the role of GABA_A_R-mediated signaling in HBV replication, the GABA_A_R agonist muscimol or the GABA_A_R antagonist picrotoxin (PTXN) were administrated to test mice. Muscimol has a distinct structural similarity with the mammalian neurotransmitters GABA, and has proved to be a potently selective agonist at GABA_A_R (Johnston, 2014). In contrast, PTXN (a mixture of picrotoxinin and picrotin) is a commonly-used noncompetitive antagonist of GABA_A_R. Notably, PTXN has been used as an antidote in barbiturates poisoning (Olsen, 2006). In order to verify whether increasing GABA signaling in liver macrophages promotes HBV replication, we used clodronate-liposome to deplete liver macrophages in HBV-carrier mice prior to GABA administration. Encapsulated in liposomes, clodronate can specifically target phagocytes cells. Liposomes are quickly recognized and swallowed by macrophages, thus permitting the clodronate concentration to an effective threshold, which triggers the apoptosis of macrophages (Moreno, 2018). We found that administration of GABA or the GABA_A_R agonist muscimol to the HBV-carrier mice increases the percentages of F4/80^+^ liver macrophages. Remarkably, GABA or muscimol promotes HBV replication. The GABA-promoted HBV replication in HBV-carrier mice was significantly reduced by the GABA_A_R antagonist PTXN or by macrophage depletion by liposomal clodronate. Collectively, our data showed that liver macrophages involve in the GABA_A_R-mediated increase of HBV replication.

### 2.2 Activation of GABA_a_R signaling enhances M2-polarization of liver macrophages

How does GABA_A_R activation promote HBV replication in hepatocytes? Our *in vitro* and *in vivo* studies revealed that GABA or muscimol treatment promotes M2-like polarization of liver macrophages in HBV-carrier mice. Remarkably, macrophage depletion by liposomal clodronate decreased the degree of the GABA-induced increase of HBV replication. Whereas adoptive transfer of CD11b^+^F4/80^+^ liver macrophages isolated from GABA-treated donor HBV-carrier mice into liposomal clodronate-treated recipient HBV-carrier mice restored HBV replication. Taken together, our data support the notion that activation of GABA_A_R signaling promotes HBV replication by enhancing M2-polarization of liver macrophages.

### 2.3 Stimulating GABA_A_Rs in macrophages causes Ca^2+^ entry

How does GABA_A_R activation promote macrophage M2 polarization? Our GSEA analyses of the GEO dataset (GSE113999) (Kim et al., 2018) revealed enrichment of Voltage-Gated Calcium Channel (VGCC) subunits in bone marrow-derived macrophages. A previous study has reported that increased intracellular Ca^2+^ is crucial for macrophage activation, migration, phagocytosis and secretion (Vaeth et al., 2015). In addition, a recent study revealed that GABA_A_R-dependent Ca^2+^ flux contributes to autophagy and phagosomal maturation in macrophages (Kim et al., 2018). In line with these reports, our results showed that activation of GABA_A_Rs increased intracellular Ca^2+^ level in primary F4/80^+^CD11b^+^ liver macrophages, as well as the calpain activity in PMA-primed THP1 cells. Given that activation of GABA_A_Rs in non-neuronal cells, such as endocrine cells (Dong et al., 2006), intestinal stem cells (Zhang et al., 2022), and epithelial cells (Xiang et al., 2007), often causes Cl^−^ efflux resulting in membrane depolarization, we propose that stimulating GABA_A_Rs in macrophages causes membrane depolarization thus activates VGCC-mediated Ca^2+^ entry, ultimately regulating M2 polarization.

### 2.4 Hepatocyte GABA signaling may not play a role in HBV replication

Studies, including ours, show that a functional GABA signaling also occurs in hepatocytes, which has protective effects against liver injury (Norikura et al., 2007; Rohbeck et al., 2023; Wang et al., 2017). For example, GABA improves mitochondrial function (Hata et al., 2019) and attenuates endoplasmic reticulum stress response (ERSR) (Shuanglian Wang, 2017), thus reducing apoptotic cell death in mice with severe acute liver injury. Moreover, the novel positive allosteric modulator of the GABA_A_ receptor, HK4, has a hepatoprotective effect on cell apoptosis induced by lipotoxicity (Rohbeck et al., 2023). Activation of GABA_A_Rs in hepatocytes induces cell-cycle activation, arrests hepatocytes at the gap 2 (G2) phase of the cell cycle and reduces chromosomal abnormalities, and decreases malignancy potential (Hata et al., 2019). Of note, emerging studies reveal that GABAergic signaling is involved in tumorigenesis as well as regulating tumor immunity, including liver hepatocellular carcinoma (Yang et al., 2023). Available data suggest that hepatocyte GABA signaling may play a critical role in liver homeostasis. However, our results showed that GABA or muscimol treatment had little effect on HBV replication in HBV-infected hepatocytes, suggesting that GABA promotes HBV replication is not by increasing the susceptibility of hepatocytes to HBV infection.

## 3 Conclusion and future perspective

Collectively, results from our recent studies (Bao et al., 2023) lead to two major conclusions: 1) liver macrophages express functional GABA signaling components; and 2) activation of GABA_A_R signaling advances M2-polarization thus promoting HBV replication.

Although activation of GABA_A_Rs in macrophages contributes to M2-polarization, GABA signaling in other types of cells may also contribute to M2 polarization. It is known that GABA_A_Rs are expressed in T-lymphocytes (Dionisio et al., 2011; Mendu et al., 2012) and the phenotypes of T-lymphocytes critically controls macrophage phenotypic polarization (Lawrence and Natoli, 2011). Given that the GABA_A_R signaling in CD4^+^ T-lymphocytes constrains Th17 development but promotes T-regulatory (Treg) cells differentiation (Kang et al., 2022), and that Tregs may limit viral control (Veiga-Parga et al., 2013), whether GABA signaling advances Treg thus promoting M2 polarization hence HBV replication requires further studies.

High-concentration GABA molecules from food-intake and intestinal bacteria are absorbed by the intestinal epithelia and get into the liver *en route* the portal vein. Under physiological conditions, most GABA molecules are up-taken into the hepatocytes by GABA transporters, maintain a relatively low and stable concentration in the liver and systemic circulation. In conditions of certain diseases such as altered intestinal microbiota and/or liver injuries, the concentration of GABA increases significantly in the liver (Petty and Schlesser, 1981) thus fundamentally affects phenotypic development of macrophages and T-lymphocytes in the organ. Therefore, the influence of GABA on HBV replication under related disease conditions also needs to be further investigated.

## Ethics approval and consent to participate

All animal procedures were performed in accordance with the Guidelines for Care and Use of Laboratory Animals of Shandong First Medical University (Numbers: ECSBMSSDU2018-2 and MECSDUMS 2018-2).

## Consent for publication

The Authors confirm: that the manuscript has not been published before; that it is not under consideration for publication elsewhere; that its publication has been approved by all co-authors, if any;

## Funding

This work is supported by grants from 10.13039/501100001809National Natural Science Foundation of China (grant number: 81873865), the 10.13039/501100007129Natural Science Foundation of Shandong Province (grant numbers: ZR2019MH066), and the Shandong First Medical University Talent Introduction Funds (to Shuanglian Wang).

## CRediT authorship contribution statement

**Yunling Chen:** Writing – original draft. **Zhaoqing Yin:** Writing – original draft. **Xiaonan Zhang:** Writing – original draft. **Yiwei Zhao:** Resources. **Tinghao Liu:** Methodology. **Wei-Yang Lu:** Writing – review & editing. **Shuanglian Wang:** Funding acquisition, Writing – original draft, Writing – review & editing.

## Declaration of competing interest

None.

## Data Availability

Data will be made available on request.
